# A biologically competitive 21 days hypofractionation scheme with weekly concomitant boost in breast cancer radiotherapy feasibility acute sub-acute and short term late effects

**DOI:** 10.1186/1748-717X-5-111

**Published:** 2010-11-22

**Authors:** Marina Guenzi, Stefano Vagge, Ngwa Che Azinwi, Alessia D'Alonzo, Liliana Belgioia, Stefania Garelli, Marco Gusinu, Renzo Corvò

**Affiliations:** 1 Department of Radiation Oncology, Istituto Nazionale per la Ricerca sul Cancro, Genoa, Italy; 2 Department of Medical Physics, Istituto Nazionale per la Ricerca sul Cancro, Genoa, Italy; 3 Università degli Studi di Genova, Italy

## Abstract

**Background:**

Radiation therapy after lumpectomy is a standard part of breast conserving therapy for invasive breast carcinoma. The most frequently used schedule worldwide is 60 Gy in 30 fractions in 6 weeks, a time commitment that sporadically may dissuade some otherwise eligible women from undertaking treatment. The purpose and primary endpoint of this perspective study is to evaluate feasibility and short-term late toxicity in a hypofractionated whole breast irradiation schedule.

**Methods:**

Between February and October 2008 we treated 65 consecutive patients with operable invasive early-stage breast cancer with a hypofractionated schedule of external beam radiation therapy. All patients were assigned to 39 Gy in 13 fractions in 3 weeks to the whole breast plus a concomitant weekly boost dose to the lumpectomy cavity of 3 Gy in 3 fractions.

**Results:**

All the patients had achieved a median follow up of 24 months (range 21-29 months). At the end of treatment 52% presented grade 0 acute toxicity 39% had grade 1 and 9% had grade 2. At 6 months with all the patients assessed there were 34% case of grade 1 subacute toxicity and 6% of grade 2. At 12 months 43% and 3% of patients presented with clinical grade 1 and grade 2 fibrosis respectively and 5% presented grade 1 hyperpigmentation. The remaining patients were free of side effects. At 24 months, with 56 assessed, just 2 patients (3%) showed grade 2 of late fibrosis.

**Conclusions:**

The clinical results observed showed a reasonably good feasibility of the accelerated hypofractionated schedule in terms of acute, subacute and short-term late toxicity. This useful 13 fractions with a concomitant boost schedule seems, in selected patients, a biologically acceptable alternative to the traditional 30 days regime.

## Background

Radiation therapy after lumpectomy is a standard part of breast conserving therapy for invasive breast cancer as it has been shown that besides significantly reducing the risk of local recurrence, it impacts favorably on patient survival [1,2]. The generally recognized standard and the most frequently used schedule worldwide is 60 Gy, delivered in 30 fractions of 2 Gy over 6 weeks, a time commitment that otherwise may generate discomfort in some women eligible for Breast Conserving Therapy (BCT). The possibility of delivering postoperative radiation therapy in a shorter period of time could circumvent this problem and result in a dramatic reduction of the nuisance factor for these patients. It would also contribute to a far more judicious use of resources and time in some busy Radiation Oncology department. The results of retrospective studies of hypofractionated radiotherapy in early breast cancer suggest satisfactory outcomes in terms of tumor control and late adverse effects [3-5]. Recent randomized trials have confirmed that hypofractioned whole-breast irradiation is equivalent to more conventional whole-breast irradiation with respect to local recurrence and cosmetic outcome [6-8]. In order to intensify treatment, a simultaneous boost dose, concomitant or integrated, has been introduced in clinics by using 3-D conformal radiotherapy or intensity-modulated radiotherapy [9,10]. Preliminary results from experiences where a boost dose was delivered either daily after whole-breast irradiation (WBI) [7] or weekly appear interesting, with reasonably good feasibility in terms of acute toxicity [11,12]. The purpose and primary endpoint of this study was to evaluate the feasibility and the acute, subacute and short term late toxicity of a hypofractionated three weeks whole breast irradiation schedule with the addition of a concomitant boost dose delivered to the tumor bed once-a-week in patients with early breast cancer submitted to lumpectomy and sentinel node dissection.

## Methods

### Patients

Sixty-five consecutive patients with operable invasive early-stage breast cancer were treated at the National Institute for Cancer Research at Genoa with hypofractionated External Beam Radiation Therapy (EBRT) as part of their BCT between Februarys to October 2008. All eligible patients had stage I-II breast carcinoma as defined by the international Union Against Cancer (fifth edition) and had gone through macroscopic total resection of the primary tumor and sentinel node biopsy. Three patients had positive or close margins because they refused to undergo re-excision, that we usually require, where possible, to obtain margins of at least 2 mm. They were nonetheless included in the protocol after due risk cautioning. Patient demographics, disease characteristics and therapy are displayed in the table 1. Patients were excluded from the study if they presented any of the following conditions: evidence of distant metastasis, presence of serious co-morbidities that could preclude radiotherapy such as cardiovascular or psychiatric disorders, tumor greater than 5 cm in its largest dimension, presence of more than 3 positive nodes, macroscopically positive margins, age less than 55 years initially, the presence of active connective tissue disease and a history of previous irradiation to the chest wall. Patients with large breasts (as defined by a cup size separation of greater than 25 cm, that is, the breast measured more than 25 cm left to right at its widest part) were also excluded [8,9]. All patients duly provided written informed consent before being assigned to treatment. Therapy was planned immediately after Breast Conserving Surgery (BCS) in low-risk patients or sequentially after systemic chemotherapy (CT) in those at higher risk of failure. Prognostic classes were assigned according to the St. Gallen Consensus Conference [13]. This protocol have been submitted and approved by our institutional ethics committee.

**Table 1:** Patient demographics, disease characteristics and therapy.

**Number of patients**	N = 65	**Surgical margins**	
**Mean age (range) in yrs**	69(53 - 86)	Negative	62 (95%)
**Tumour class(AJCC)**		Positive	1 (2%)
pTis	3 (5%)	Close	2 (3%)
pT1a	4 (6%)	**Hormonal status**	
pT1b	10 (15%)	HR positive	60 (92%)
pT1c	34 (52%)	HR negative	5 (8%)
pT2	14 (22%)	**Hormone therapy**	
**Max tumour diam. (range) mm**	3 - 30	Yes	57 (88%)
**Grading**		No	5 (13%)
G1	12 (18%)	**Chemotherapy**	9 (14%)
G2	39 (60%)		
G3	14 (22%)		
**Proliferative index (Ki67) %**			
≤ 15	39 (60%)		
> 15	26 (40%)		
**Nodal status**			
pN0	58 (89%)		
pN1(a)	7 (11%)		

### Radiation fractionation and treatment

**Figure 1:**
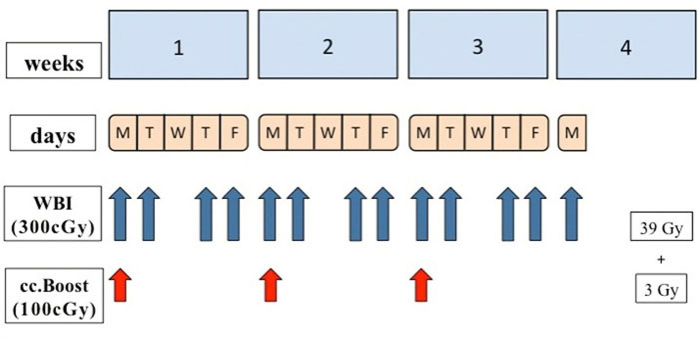
**Fractionation scheme**. **m: **monday; **t: **tuesday; **w: **wednesday; **t: **thursday; **f: **Friday. **WBI: **whole breast irradiation. **cc.boost: **concomitant boost

The basic scheme of treatment consisted in the delivery of 39 Gy in 13 fractions 4 times a week to the whole breast plus a once weekly concomitant boost dose of 1 Gy to the lumpectomy area immediately after whole breast irradiation (WBI) (thus a total boost dose of 3 Gy in 3 fractions once a week). Doses were prescribed to international reference points. Total treatment time was 3 weeks plus 1 day, and the total nominal dose to the lumpectomy area (considering the cumulative dose to the whole breast and to the surgical bed) was 42 Gy. Generally, weekly treatment would start on Monday and end on Friday with a pause planned for Wednesday. The boost dose was added on Monday (Figure 1). Portal films of the whole breast were taken at least once during the first day of irradiation and compared with Digitally Reconstructed Radiographs (DRR) for matching. The ethic committee of our institution approved the final protocol.

### Radiobiological equivalent dose

Using the Linear-quadratic cell survival model [equation 1, *appendix*] we calculated Biologically Equivalent Doses (BEDs) for the breast and boost volumes [14]. For this calculation we assumed an *α*/*β *ratio of 4 Gy for tumor response [15], 10 Gy for acute responding normal tissues [16], 1.7 Gy for late-responding tissues (fibrosis) [17] and 2.5 Gy for vascular damage [18]. The biological comparison between the standard and the explored RT schedule is shown in table 2. Although the BED for cancer clonogens was equivalent for the 42 Gy in 13 fractions schedule, we hypothesized that this similar dose equivalence could be advantageous for our schedule by the greater microvascular dysfunction on the boost site that the higher dose per fraction could achieve. It may be worth noting that this factor of tumor kill is normally not included in mathematical models for BED calculation.

**Table 2:** BED comparison between standard and explored RT schedule

RT schedule	BED tumor control *α*/*β*_4_		BED acute effects *α*/*β*_10_		BED fibrosis *α*/*β*_1.7_		BED vascular damage *α*/*β*_2.5_	
W.B. = whole breast B.S. = tumor bed side	W.B.	B.S.	W.B.	B.S.	W.B.	B.S.	W.B.	B.S.

*60 Gy/30 F/6 W* *(50 Gy + 10 Gy seq.boost)*	75	90	60	72	109	131	90	108

*50 Gy/25 F/5 W* *(no boost)*	75	75	60	60	109	109	90	90

*42 Gy/13 F/3W + 1 day* *(39 Gy + 3 Gy cc.boost)*	**68**	**77**	**51**	**56**	**108**	**123**	**86**	**97**

*52 Gy/20/F/5 W* *(46 Gy + 6 Gy cc.boost)*	72	87	57	66	108	135	88	108

*UK START TRIAL A* *41.6 Gy/13 F/5 W*	75	75	55	55	120	120	95	95

*UK START TRIAL A* *39 Gy/13 F/5W*	68	68	51	51	108	108	86	86

boost = concomitant boost; seq.boost = sequential boost; F = fractions; W = weeks

### Volumes of interest and treatment planning

**Figure 2:**
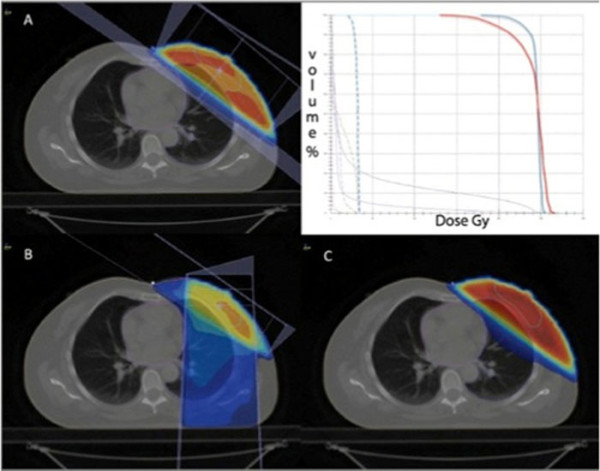
**An example of a sum plan and Dose Volume Histogram**. **A**: whole breast; **B**: boost; **C**: plan sum

A planning CT scan was carried out for each patient with the patient positioned supine on a "wing-board" with both arms raised above the head. Radiopaque wires and markers were used to locate palpable breast tissue and visible surgical scars. Three tattoos were made on the thoracic skin to enable patient repositioning during treatment. The CT scans went from the level of the larynx to the upper abdomen with both lungs included. Scan thickness was 10 mm. The Whole Breast Clinical Target Volume (WB-CTV) included glandular breast tissue and did not extend to cover the pectorals major, the ribs or the skin. The Whole Breast Planning Target Volume (WB-PTV) was generated by the addition of a 3-D 3-5 mm margin around the WB-CTV where possible considering the presence of nearby organs at risk (OARs) while for the cranial and caudal directions a 10 mm margin was used. The definition of the lumpectomy cavity was guided by the presence of surgical clips, hematoma, seroma or other surgery-induced changes considered to be part of the cavity. The boost CTV was generated by adding at least a 2 mm margin around the lumpectomy cavity and the corresponding PTV created by adding a further 2 mm 3 D margin. The heart and ipsilateral lung were considered OARs. The heart was contoured from the pulmonary trunks superiorly to its base and included the pericardium. The major blood vessels were excluded. The ipsilateral lung was contoured in all its extension. Three Dimensional Conformal Radiotherapy (3DCRT) plans were generated using either of two TPS systems (CMS Xio or Varian Eclipse). Treatment plans for the whole breast were generated using two opposed tangential beams. Beam weighting, gantry angles, wedges, multi leaf collimator (MLC) shielding and beam energies were determined to achieve optimal dose conformity and distribution as well as maximal avoidance of the heart and ipsilateral lung. The boost plan consisted of two or more photon beams suitably angled and optimized by the use of wedges and selective MLC shielding. Both plans (Whole breast and Boost) aimed for a 95% isodose level encompassing the PTVs and plan evaluation was enhanced by the use of Dose Volume Histograms (DVHs) and a chosen Conformity Index (CI). An example of a sum plan and DVH are displayed in figure 2.

### Follow up

Clinical checks were carried out halfway through treatment. Follow up for acute toxicity was arranged at treatment end and at 3 months. Baseline mammography was planned at 8 months after completion of treatment and yearly thereafter. Acute toxicities were graded based on the RTOG acute toxicity scale [19] (table 3). Subacute and late toxicities were graded using the Modified LENT SOMA scoring system [20] (table 4) and was assessed at 6 months, at 12 months and thereafter planned every six months. The toxicity parameters examined included the following: erythema, breast edema, desquamation, ulceration, fibrosis, telangiectasia, hyperpigmentation, retraction and atrophy.

**Table 3:** RTOG Acute Skin Score

**Grade 0**	No change over baseline
**Grade 1**	Follicular, faint or dull erythema/epilation/dry desquamation/decreased sweating
**Grade 2**	Tender or bright erythema, patchy moist desquamation/moderate edema
**Grade 3**	Confluent, moist desquamation other than skin folds, pitting edema
**Grade 4**	Ulceration, haemorrhage, necrosis

**Table 4:** Modified LENT SOMA Scale

	Grade1	Grade 2	Grade 3	Grade 4
**Fibrosis**	Barely palpable increased density	Definite increased density and firmness	Very marked density, retraction and fixation	
**Telangiectasia**	< 1cm^2^	1cm^2 ^- 4cm^2^	> 4cm^2^	
**Hyperpigmentation**	Mild	Moderate	Severe	
**Retraction/Atrophy**	10 - 25%	> 25 - 40%	> 40 - 75%	Whole breast
**Ulcer**	Epidermal only, ≤ 1cm^2^	Dermal, > 1cm^2^	Subcutaneous	Bone exposed, necrosis

## Results

At the time of reporting, 65 patients had achieved a minimum follow up of 21 months (median FU 24 months, range 21-29 months). All accrued patients were included in this analysis. The mean PTV of the whole breast volume was 642 cc (range 319-1198 cc), the mean PTV of the boost volume was 57 cc (range 21-148) and the mean ratio between the whole breast and boost volume in percentage was 9% (range 3-20 cc). At the end of treatment and until the first 3 months the majority of patients were free of noteworthy acute toxicity, just the 9% of them presented bright erythema (table 5). The evaluation of subacute toxicity at 6 months showed a grade 2 barely in 4 patients (6%). Mild hyperpigmetation have been detected in 22 (34%) patients, the rest, 39 (60%) were toxicity free (table 6). At 12 months, with all patients assessed, 28 (43%) and 2 patients (3%) presented with clinical grade 1 and grade 2 fibrosis respectively while 3 patients (5%) presented grade 1 hyperpigmentation (table 6). At 24 months grade 2 late fibrosis was present just in 2 patients (3%) o 56 evaluable (table 6).

**Table 5:** Acute toxicity assessment (based on RTOG acute skin scoring)

	G0	G1	G2	G3	N^0 ^of patients
**Treatment end**	34 (52%)	25 (39%)	6 (9%)	0	65
**3 months**	40 (62%)	19 (29%)	6 (9%)	0	65

**Table 6:** Late toxicity assessment (based on Modified LENT SOMA)

	G1	G2	G3	G4	N^0 ^of patients
**At 6 months (subacute)**					
***Hyperpigmentation***	22 (34%)	4 (6%)	0	0	65
**At 12 months**					
***Fibrosis***	28 (43%)	2 (3%)	0	0	65
***Hyperpigmentation***	3 (5%)	0	0	0	65
At 24 months*					
***Fibrosis***	25 (45%)	2 (3%)	0	0	56
***Hyperpigmentation***	0	0	0	0	56

* A total of 56 patients seen at 24 months or more with 29 (52%) free of side effects.

## Discussion

Radiotherapy after lumpectomy improves local control and overall survival [2] and it is considered part of the conservative treatment. Standard radiation requires daily treatment for 6 to 7 weeks and this may be a serious inconvenience for many patients, especially for the elderly. Delivering postoperative radiation therapy in a shorter period of time could result in a significant reduction of this problem for patients. Shorter radiation schedules based on radiobiological models offer the promise of equivalent local control to standard radiation therapy by giving larger doses per fraction in shorter periods of time [21]. Several experiences and results of randomized trials have been reported and offer encouraging outcomes. Recently Whelan et al examined whether a 22-day radiation therapy fractionation schedule was as effective as the more traditional 35-day schedule in reducing recurrence in 1234 women with invasive breast cancer who underwent BCS with pathologically clear resection margins and negative axillary lymph nodes. The patients were randomly assigned to receive whole breast irradiation of 42.5 Gy in 16 fractions over 22 days (short arm - 622 pts) or whole breast irradiation of 50 Gy in 25 fractions over 35 days (long arm - 612 pts). With a median follow-up of 12 years no difference in local recurrence, disease-free or overall survival rates and cosmetic outcome was detected between study arms. They conclude that the more convenient 22-day fractionation schedule appears to be an acceptable alternative to the 35-day schedule [8]. The START A (Standardization of Breast Radiotherapy) from the UK trial [6] has shown that 41.6 Gy/13 fractions or 39 Gy/13 fractions are similar to the control regimen of 50 Gy/25 fractions in terms of local-regional tumor control and late normal tissue effects, a result consistent with the results of START trial B [7], which has shown that a radiation schedule of 40 Gy/15 fractions offers equivalent results to the standard schedule of 50 Gy/25 fractions. Fujii et al. [22], from Kawasaki Medical School in Japan, in a prospective study have reported early toxicity and treatment results of a total of 248 patients (251 breasts) treated with a shorter fractionation regimen. The whole breast was irradiated with a total dose of 42.5-47.8 Gy in 16-20 fractions. Patients with positive margins received an additional boost irradiation to the tumor bed of 10-13.3 Gy in 4-5 fractions using 4-11 MeV electrons. With a median follow-up time of 26 months radiation dermatitis was observed in 221 patients (207 patients with grade 1, 14 with grade 2): they conclude that that shorter fractionation of RT following BCS has acceptable acute morbidity and can obtain a reasonably good cosmetic outcome. Livi et al [23] evaluated the incidence of locoregional recurrence and the cosmetic results in a group of 539 patients with breast cancer treated with a hypofractionated schedule of adjuvant radiotherapy after conservative surgery. The dose delivered was 44 Gy (2.75 Gy daily fraction). The tumor bed boost (10 Gy) was administered by the use of electrons. They obtain a low local relapse rate and good tolerance (late toxicity: 76.4% pts or grade 0-1, 20.9% pts grade 2, 2.5% pts grade 3. No patients developed grade 4 toxicity). They conclude that this approach resulted in an effective treatment in terms of local control in patients with negative or one to three positive axillary nodes and negative surgical margins. Patients treated with a hypofractionated schedule showed very good cosmesis. Through empiric observation, it has become clear that the therapeutic ratio, the balance between tumor cell kill and normal tissue damage, is affected not only by fraction size but also the total dose of radiation and in some instances overall treatment time and the volume of tissue irradiated. Radiobiological models have been developed in an attempt to predict improvement in the therapeutic ratio through manipulation of these different variables. The most commonly used model is the linear-quadratic equation; it predicts that the biological effect of radiation will be directly proportional to total dose and fraction size. Based on the results of some important randomized trials [6-8], from February 2007 we began treating early stage breast cancer patients using a hypofractionated schedule of 46 Gy prescribed to the ICRU 50 reference point dose and delivered in 20 fractions, 4 times a week for 5 weeks. Once a week, immediately after whole breast irradiation, a concomitant photon boost of 1,2 Gy was delivered to the lumpectomy area. Corvò et al. [12] already published their experience and found this schedule to be well tolerated, without important acute toxicity. On this basis, in an attempt to intensify treatment using a more hypofractionated radiotherapy scheme and a weekly simultaneous boost, we began a phase two study. The basic course consisted of 39 Gy prescribed to the ICRU 50 reference point dose and delivered in 13 fractions, 4 times a week for 3.1 weeks. Once a week, immediately after whole breast irradiation, a concomitant photon boost of 1 Gy was delivered to the lumpectomy area.

Using the classic linear-quadratic cell survival model [equation 1, *appendix*] we calculated the Biological Equivalent Doses (BED) for the standard radiotherapy and hypofractionated schedules. We then attempted a BED comparison between the schemes. Based on recent investigations, an *α*/*β *value of 4 Gy was assumed for tumor control, which is quite close to that estimated for late responding tissues [15]. To compare the effectiveness of schedules consisting of different total doses and doses per fraction we convert each schedule into an equivalent schedule of 2 Gy fractions that would give the same biological effect [equation 2, *appendix*][14]. The values calculated are reported in table 3. Our shorter fractionation regiment (42 Gy/13fx/21 days) came out as equivalent to 77 Gy, on the tumor bed, given by way of the standard schedule. None of the comparisons assessed the influence of the time factor on the value of the equivalent doses. Calculating BED [equation 3, *appendix*] were time is taken into account as an independent variable [21], our more hypofractionated schedule again turns out to be similar or actually compares favorably, in terms of acute effects and tumor control, with the standard regimen as well as with the UK START TRIAL A schemes (table 7). The vascular damage was calculated on the basis of the *α*/*β *ratio of capillary component [18] with the hypothesis that the microvascular dysfunction induced by radiation [24] should be advantageous for clonogenic cell control on the tumor bed.

**Table 7:** BED comparison considering total treatment time for different schedules

RT schedule	BED tumor control *α*/*β*_4_		BED acute effects *α*/*β*_10_		BED fibrosis *α*/*β*_1.7_		BED vascular damage *α*/*β*_2.5_	
W.B. = whole breast B.S. = tumor bed side	W.B.	B.S.	W.B.	B.S.	W.B.	B.S.	W.B.	B.S.

*60 Gy/30 F/6 W* *(50 Gy + 10 Gy seq.boost)*	68	78	53	60	109	131	90	108

*50 Gy/25 F/5 W* *(no boost)*	68	68	53	53	109	109	90	90

*42 Gy/13 F/3W + 1 day* *(39 Gy + 3 Gy cc.boost)*	**68**	**77**	**51**	**56**	**108**	**123**	**86**	**97**

*52 Gy/20/F/5 W* *(46 Gy + 6 Gy cc.boost)*	65	75	49	54	108	135	88	108

*UK START TRIAL A* *41.6 Gy/13 F/5 W*	68	68	48	48	120	120	95	95

*UK START TRIAL A* *39 Gy/13 F/5W*	61	61	43	43	108	108	86	86

boost = concomitant boost; seq.boost = sequential boost; F = fractions; W = weeks

## Conclusions

The purpose and primary endpoint of this study was to determine the acute toxicity and feasibility of a course of radiation administered in hypofractionation. The clinical results observed in 65 consecutive patients with a median follow-up 24 months (range 21 - 29 months) demonstrated a reasonably good feasibility of the schedule in terms of acute and subacute toxicity as well as in terms of compliance to treatment. The initial analysis of late effects appears equally promising. At the moment this more convenient 13 fraction schedule seems an acceptable alternative to the traditional 30 day regime. Longer follow-up is being arranged to confirm these results and to evaluate whether this schedule assures excellent local-regional disease control besides good tolerability. If that turns out to be the case, our results would be in line with the results of other important studies in the literature which indicate a significant improvement in patient quality of life through the reduction of total treatment time while guaranteeing acceptable late effects and local control endpoints. Furthermore, a reduction of such magnitude in treatment duration would possibly allow for a far more efficient use of healthcare resources.

## Appendix

Equation 1

$$BED=D\left(1+\frac{d}{\alpha /\beta }\right)$$

where:

D: total dose delivered in Gy

d: the size of fractions in Gy

Equation 2

$$LQED_{2}=D\left(\frac{\frac{\alpha }{\beta }+d}{\frac{\alpha }{\beta }+2}\right)$$

where:

LQED_2_: is the biologic equivalent of a total dose in 2Gy fractions.

d: is the size of fractions in Gy

Equation 3

$$LQED_{2}=D\left(\frac{\frac{\alpha }{\beta }+d}{\frac{\alpha }{\beta }+2}\right)$$

where:

T: overall time of radiotherapy (days, with first day counted as Day 0)

Tk: onset (Kick-off) time of repopulation in the tissue of interest: 21 days

*α*: radiosensitivity coefficient of non-recoverable damage: 0.27 Gy

Tp: potential doubling time of cancer repopulating cells = 3 days

## Competing interests

The authors declare that they have no competing interests.

## Authors' contributions

RC, MG* carried study design. MG*, NCA, SV collected the data and performed statistical analysis and drafted the manuscript. AD, LB, SV, NCA took care of the patients and helped to draft the manuscript. SG, MG: performed treatment plans and gave advice on the work. All authors have read and approved the final manuscript.

MG*: Marina Guenzi
